# Extranodal Rosai–Dorfman Disease Presenting as a Mediastinal Mass with Pulmonary Artery Invasion

**DOI:** 10.1155/2018/3915319

**Published:** 2018-04-12

**Authors:** Andrew R. Orr, Daniel Lefler, C. Deshpande, Pallavi Kumar

**Affiliations:** ^1^Department of Internal Medicine, Hospital of the University of Pennsylvania, Philadelphia, PA, USA; ^2^Department of Pathology, Hospital of the University of Pennsylvania, Philadelphia, PA, USA; ^3^Department of Internal Medicine, Division of Hematology and Oncology, Hospital of the University of Pennsylvania, Philadelphia, PA, USA

## Abstract

Rosai–Dorfman disease (RDD) is a rare, nonmalignant disorder of histiocyte proliferation typically involving the cervical lymph nodes. However, a subset of patients with RDD will display extranodal manifestations that are highly variable in presentation, more challenging to diagnose, and less likely to spontaneously regress compared to nodal disease. While case reports of extranodal involvement in nearly every organ system exist, documented instances of mediastinal and pulmonary artery involvement are particularly rare. This study describes the case of a middle-aged woman presenting with new onset right heart failure who was found to have extranodal RDD in the form of a large mediastinal mass with invasion and occlusion of the main pulmonary arteries.

## 1. Introduction

First recognized as a clinical entity in 1969, Rosai–Dorfman disease (RDD), or sinus histiocytosis with massive lymphadenopathy, is a rare, nonmalignant disorder of histiocyte proliferation typically involving the cervical lymph nodes [1]. It belongs to the group of primary histiocytic disorders including Langerhans cell histiocytosis and Erdheim–Chester disease and is ultimately differentiated from these disorders based on its characteristic expression of cellular markers and demonstration of emperipolesis, or histiocytic consumption of lymphocytes, on histologic analysis [2, 3]. While associations with viral disorders and IgG4-mediated disease have been suggested, no definitive etiology has been identified [2, 4]. There are approximately 600 reported cases of RDD, but the true number of patients with RDD remains unknown [5].

Although classically confined to lymph nodes, 23–40% of patients with RDD manifest extranodal involvement, either isolated or concurrent with lymphadenopathy [6]. Extranodal RDD most commonly presents as a painless, palpable mass and appears to possess distinct features when compared to nodal disease. In contrast to nodal disease, extranodal disease affects females more frequently, with one case series of extranodal disease documenting a 90% female predominance [7]. Further, while nodal disease often spontaneously regresses, the course of extranodal disease is generally less indolent and can be aggressive if vital organs are involved, with one case series documenting a mortality rate of 45% [2]. In the largest review of extranodal RDD by Gaitonde, the most frequent extranodal sites were found to be skin and soft tissue (16%); nasal cavity and paranasal sinuses (16%); eye, orbit, and ocular adnexa (11%); bone (11%); salivary gland (7%); central nervous system (7%); oral cavity (4%); kidney and genitourinary tract (3%); respiratory tract (3%); liver (1%); tonsil (1%); and breast (<1%) [8]. Finally, pathologic diagnosis of extranodal RDD is complicated by more pronounced fibrosis and less conspicuous lymphocytophagocytosis compared to nodal disease [6].

## 2. Case Report

We report the case of a 41-year-old woman with a history of presumed spinocerebellar ataxia who presented with right heart failure from an invasive mediastinal mass with near complete occlusion of her pulmonary arteries.

**Figure 1:**
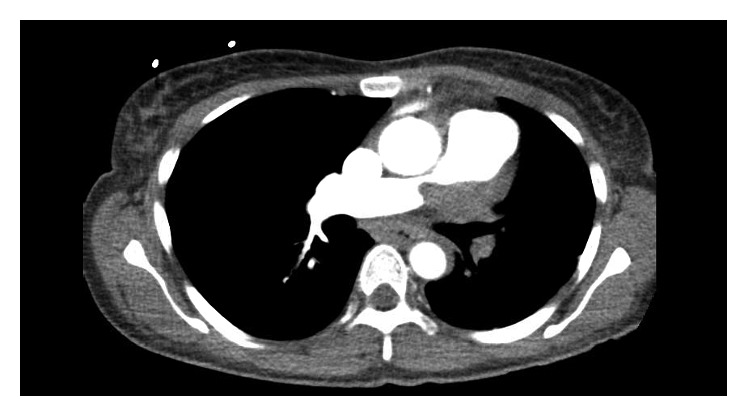
CT scan showing mediastinal mass narrowing the right pulmonary artery with full occlusion of the left pulmonary artery.

She was in her usual state of health until first presenting to the emergency department in February 2017 with bilateral lower extremity edema and progressive dyspnea on exertion. She was found to have an amorphous, invasive superior left mediastinal mass involving the main and left pulmonary arteries with occlusion of the left pulmonary arterial tree and severe narrowing of the right pulmonary artery (Figure 1). At that time, she was noted to have moderate cardiomegaly with right heart enlargement and findings of elevated right heart pressures as well as several scattered, small nonspecific pulmonary nodules. Subsequent PET scan showed marked fluorodeoxyglucose (FDG) avidity of the anterior mediastinal mass. She underwent intravascular biopsy with interventional radiology during that first admission, and an arteriogram revealed a severely stenosed but still-patent right main pulmonary artery with no filling of the left main pulmonary artery. She was discharged with subspecialist follow-up pending biopsy results. However, initial pathology revealed only thrombus material and a minute fragment of unremarkable intima. The patient was unable to tolerate the scheduled cardiac MRI for repeat biopsy planning and was lost to follow-up until representing to thoracic surgery clinic in May 2017 after a progressive decline. At that visit, she was directly admitted for immediate further evaluation given the extent of her mass.

**Figure 2:**
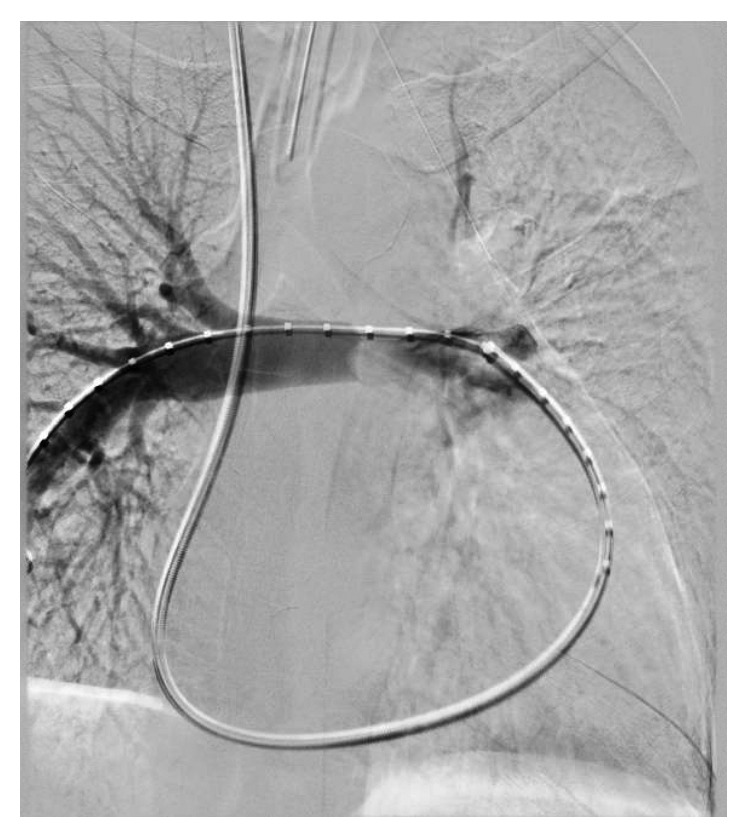
Fluoroscopic view showing stenotic portion of the proximal right pulmonary artery prior to stenting.

**Figure 3:**
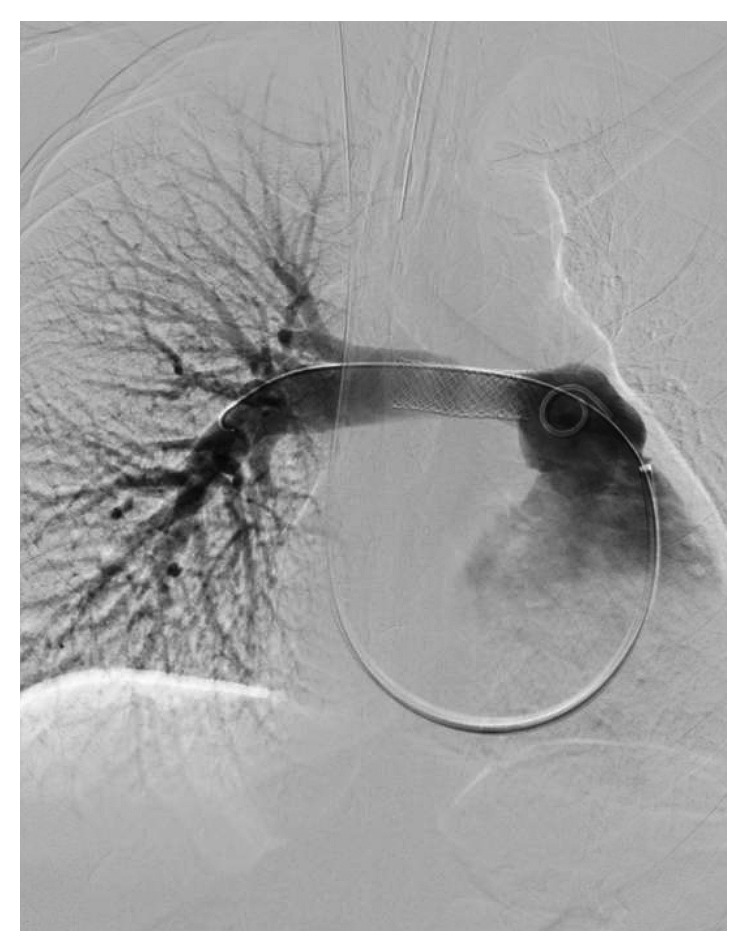
Fluoroscopic view of the right pulmonary artery showing stent across the previously stenotic area.

On admission in May 2017, her right heart function had declined considerably with echocardiogram notable for a severely dilated right atrium and severely dilated right ventricle with the interatrial septum bowing to the left, suggestive of right heart failure. She underwent a second biopsy via bronchoscopy; however, pathology was again nondiagnostic and notable only for fragments of fibrous tissue with a mixed inflammatory infiltrate. She then underwent a third biopsy via repeat intravascular approach that was also nondiagnostic despite seemingly adequate yield and notable only for vessel wall fragments with myxoid degeneration and few small crushed cells, favoring an inflammatory infiltrate. Although there was suspicion for sarcoma or lymphoma, the radiation oncology and medical oncology teams opted to defer empiric treatment until a definitive diagnosis was made. The cardiac surgery team felt that a biopsy via anterior chest wall window was too high risk due to the orientation of the mass. Due to the significant risk of impending hemodynamic instability from her mass, she underwent pulmonary artery stenting with the interventional radiology team, who felt that they would be able to safely stent the right pulmonary artery but could not intervene on the totally occluded left pulmonary artery. Her gradients improved immediately after stenting, and she tolerated the procedure without complication (Figures 2 and 3).

**Figure 4:**
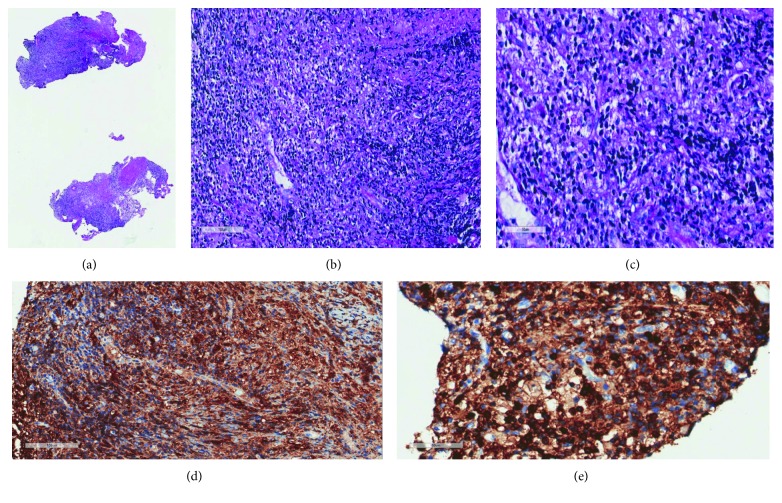
Biopsy results. (a) Hematoxylin and eosin stain (H&E) at 2x magnification–fibrosis with chronic inflammatory cell response. (b) H&E at 20x magnification–macrophages with lymphocytes and plasma cells. (c) H&E at 40x magnification–emperipolesis. (d) CD163 immunostain at 20x magnification highlighting macrophages. (e) CD45 immunostain at 40x magnification highlighting lymphocytes and emperipolesis.

A fourth biopsy taken at the time of stenting ultimately revealed fibrous tissue with lymphohistiocytic and plasma cell infiltrate with positive staining for S100, CD68, and CD163, negative staining for CD1a and Factor XIIIa, and emperipolesis, consistent with extranodal Rosai–Dorfman disease (Figure 4). In order to rule out other disease processes, pathologic analysis revealed no significant increase in IgG4+ plasma cells and no evidence of bacteria, acid fast, or fungal organisms on Gram, Fite, AFB, Grocott, and PAS stains. Importantly, she was evaluated by the genetics team, who determined it was unlikely that her development of RDD was at all related to her presumed diagnosis of spinocerebellar atrophy.

## 3. Comment

The differential diagnosis of extranodal RDD is broad and includes sarcoma, lymphoma, metastasis, fibrotic disorders such as IgG4-related disease, and chronic infections (mycobacterial or fungal) [2, 5]. Due to the absence of pathognomonic features on imaging, diagnosis of RDD relies on tissue sampling and pathologic review. As evidenced by the need for four biopsies in this case, histologic diagnosis of extranodal Rosai–Dorfman disease can be challenging due to lack of characteristic microscopic features, including emperipolesis [9].

At the time of publication, extranodal RDD as an isolated mediastinal mass has been described in only four cases and has manifested as bilateral hilar lymphadenopathy, posterior mediastinal mass, anterior mediastinal mass, and primary thymic involvement [4, 10, 11]. Cardiac involvement by RDD is similarly rare and has been cited in only 19 cases in the literature [12]. Among that series, we discovered 4 cases with pulmonary arterial involvement. Three of these patients underwent successful treatment of the lesion, while one died after cardiac arrest during invasive intravascular biopsy (Table 1) [13].

While treatment is often unnecessary in RDD because of spontaneous resolution of lymphadenopathy, intervention is required when extranodal disease compresses vital structures. The optimal treatment for extranodal RDD remains unknown. Current modalities include surgical resection, chemotherapy, and radiation. Chemotherapy is often ineffective, and usually involves one or more of the following: methotrexate, 6-mercaptopurine, 6-thioguanine, interferon-*α*, and vinca alkaloids. Radiation has mixed results, and one case series showed that surgical management induced complete remission in eight of nine patients (89%) [17]. All three of the patients with previously described pulmonary arterial involvement underwent extensive cardiothoracic surgery for resection, with favorable outcomes, consistent with the positive outcomes offered by surgical management. These cases stress the importance of obtaining definitive pathology specimens prior to operating and entertaining the possibility of RDD in cases where tissue analysis seems unrevealing.

Because she was deemed a poor surgical candidate, our patient was planned for radiation to her mediastinal disease after her pulmonary artery stenting. Chemotherapy was deferred in favor of monitoring her response to radiation therapy. Six weeks after beginning therapy, a follow-up CT showed a decrease in the infiltrative mediastinal mass reflecting partial therapeutic response, a widely patent right pulmonary artery, interval improvement of right atrial and ventricular enlargement, and stable residual pulmonary nodules consistent with previously described pulmonary manifestations of RDD [18].

There is no current consensus on treatment of RDD involving the pulmonary vasculature, as surgery is preferred in cases of involvement of other vital organs [17]. However, surgery is high-risk and can require complicated anatomic reconstruction of the great vessels, as described in the cases laid out in this review. Our case provides evidence that pulmonary artery stenting combined with radiation therapy may be a viable option for these rare patients when surgery is not possible. Further studies are necessary to more fully compare the risks and benefits of the multiple therapies currently used to treat RDD.

## 4. Conclusion

In this study, we present, to our knowledge, the fifth case of extranodal RDD manifesting as a mass with pulmonary artery involvement. While surgical resection was performed for previous patients with this condition, pulmonary artery stenting followed by radiation therapy has achieved clinical stability in this patient. Further studies are needed to determine the optimal management of this rare presentation of a rare disease.

## Figures and Tables

**Table 1:** Clinicopathologic features and treatment of RDD involving the pulmonary artery.

Reference	Clinical features	Pathologic features	Treatment	Outcome
Rehman et al. [13]	Syncope with central pulmonary artery filling defect in a 61-year-old woman	Grossly: tan, well-circumscribed mass within pulmonary trunk	N/A	Deceased during attempt at ultrasound-guided intravascular biopsy
Morsolini et al. [14]	Dry cough with FDG-avid mass infiltrating right pulmonary artery in a 62-year-old man	Grossly: white-yellow mass of solid, fleshy tissue. Microscopically: large, histiocyte-like cells with emperipolesis in background of mature plasma cells, small lymphocytes, and foamy histiocytes	Pulmonary artery endarterectomy	Stable and disease-free at 9 months
Walters et al. [15]	Progressive dyspnea and lower extremity edema with FDG-avid masses nearly completely obstructing her main pulmonary artery, right pulmonary artery and left pulmonary artery in a 22-year-old woman	Gross: Tan-white, solid, finely granular specimen. Microscopically: emperipolesis (in 5–10% of histiocytes) and histiocytic proliferation among an inflammatory infiltrate of plasma cells and lymphocytes set in a fibrous stroma	Debulking operation on cardiopulmonary bypass	No disease recurrence at 5 months from operation
Prendes et al. [16]	Progressive dyspnea on exertion with cor pulmonale and bilateral pulmonary artery narrowing due to a mediastinal mass in a 42-year-old woman	Microscopic: Two inconclusive biopsies revealing lymph and fibroadipose tissue, respectively, prior to successful biopsy with pathognomonic features of RDD	Median sternotomy with full cardiopulmonary bypass for resection and reconstruction of the great vessels with multiple grafts placed in the aorta and pulmonary artery	Normalization of right ventricular size with improved function and clinically stable without symptoms or recurrence at 12 months from operation
